# A Two-Stage Model for In Vivo Assessment of Brain Tumor Perfusion and Abnormal Vascular Structure Using Arterial Spin Labeling

**DOI:** 10.1371/journal.pone.0075717

**Published:** 2013-10-02

**Authors:** Patrick W. Hales, Kim P. Phipps, Ramneek Kaur, Christopher A. Clark

**Affiliations:** 1 Imaging and Biophysics Unit, Institute of Child Health, University College London, London, United Kingdom; 2 Neuro-oncology Department, Great Ormond Street Hospital, London, United Kingdom; University Hospital of Heidelberg, Germany

## Abstract

The ability to assess brain tumor perfusion and abnormalities in the vascular structure *in vivo* could provide significant benefits in terms of lesion diagnosis and assessment of treatment response. Arterial spin labeling (ASL) has emerged as an increasingly viable methodology for non-invasive assessment of perfusion. Although kinetic models have been developed to describe perfusion in healthy tissue, the dynamic behaviour of the ASL signal in the brain tumor environment has not been extensively studied. We show here that dynamic ASL data acquired in brain tumors displays an increased level of ‘biphasic’ behaviour, compared to that seen in healthy tissue. A new two-stage model is presented which more accurately describes this behaviour, and provides measurements of perfusion, pre-capillary blood volume fraction and transit time, and capillary bolus arrival time. These biomarkers offer a novel contrast in the tumor and surrounding tissue, and provide a means for measuring tumor perfusion and vascular structural abnormalities in a fully non-invasive manner.

## Introduction

Solid tumors account for approximately two thirds of all childhood cancers, of which brain and central nervous system tumors are the most common [1]. To grow beyond a few millimeters in size, tumors must develop networks of new vascular supply [2]. The new vessels which are formed are often tortuous [3], display increased permeability to macromolecules [4], and have significantly larger diameters [5], [6]. Characteristic changes in vessel shape occur not just at the capillary level, but include larger, initially healthy vessels, and extend over a distance beyond the tumor margin [6], [7]. Imaging modalities which are sensitive to both abnormal flow characteristics and vascular structure can therefore provide potential surrogate biomarkers for the evaluation of tumor malignancy, growth, and response to treatment.

Arterial spin labeling (ASL) is an emerging technique for fully non-invasive quantification of cerebral blood flow (CBF) [8], [9]. Although the signal to noise ratio (SNR) is inherently lower than MR-based perfusion measurements acquired using injected paramagnetic contrast agents, ASL has the advantage that it employs an endogenous tracer, by magnetically labeling water in the arterial blood supply. As such, it is a completely non-invasive and non-ionizing technique, allowing for safe repeated measurements in patients, increased patient comfort, and avoids the risk of nephrogenic systemic fibrosis associated with the use of gadolinium based contrast agents in patients with renal failure [10]. These combined benefits have seen ASL move from the field of research into routine clinical practice in recent years [11]–[13].

Perfusion quantification using ASL is performed by inverting the longitudinal magnetization of the arterial blood flowing into the tissue, waiting for a given ‘inflow time’ (TI), then acquiring an image (known as the ‘*label*’ acquisition). The same process is repeated without labeling the inflowing arterial blood (the ‘*control*’ acquisition), after which the perfusion information is contained in the subtraction between the two acquisitions (*dM*, in which *dM* =  *control – label* signal intensity). A complication associated with this technique is the presence of intra-vascular signal if the ASL acquisition is performed at short TI times, before the labelled bolus has reached the capillary bed. These appear as bright foci in *dM* images, caused by the presence of tagged blood in arterial vessels which is destined to perfuse more distal tissue. The simplest way to eliminate the intra-arterial contribution to the ASL signal is to perform the acquisition at a longer TI; however, the recovery of the inverted longitudinal magnetization of the labelled blood-water at longer TIs results in a reduction in SNR. Alternatively, bipolar crusher gradients have been employed to eliminate vascular artefacts, by dephasing the moving spins in large vessels [14], [15]. Although effective at eliminating the bright foci seen in large arteries, these techniques require prior assumptions regarding arterial flow velocity, and due to scan time constraints the crusher gradients are generally only applied in a single direction, reducing the efficiency of the technique in vessels perpendicular to the gradient direction. Furthermore, these bipolar gradients cannot be employed in conjunction with the GRadient And Spin Echo (GRASE) readout used in a number of multi-TI ASL sequences, due to conflicts with the Carr-Purcell-Meiboom-Gill echo sequence inherent in the technique [16].

When ASL acquisitions are performed over a range of TIs, the dynamic behaviour of the inflowing bolus of labelled blood can be studied. Early work by Buxton et al. [17] demonstrated a general kinetic model for the time-dependent *dM* signal, which takes into account the history of delivery of magnetization by arterial flow, and clearance by venous flow and longitudinal relaxation. This kinetic model is able to account for local differences in bolus arrival time (BAT), which not only improves the accuracy of CBF quantification, but has also been shown to be a physiologically useful parameter itself [18]–[20]. However, CBF quantification using the Buxton model is still corrupted by vascular artefacts, as it is assumed that labelled blood instantaneously exchanges with tissue upon arrival, which neglects both the finite permeability of the capillary walls, and the ‘through-flow’ of blood in large arteries destined for capillaries distal to the voxel.

Alternatively, a number of ‘two-compartment’ ASL models have been developed, which relax the assumption of ‘instantaneous tracer exchange’ by accounting for the finite permeability of the vasculature in the capillary bed [21]–[24]. Although more physiologically accurate, these models require either prior assumptions regarding the local capillary permeability-surface product (PS) and vascular volume fraction, which are unlikely to hold true under pathological conditions, or a large number of fitted parameters. Furthermore, with the exception of [23], they do not account for the ‘through-flow’ effect in voxels containing large arterial vessels.

An alternative approach was presented recently by Chappell et al. [25], in which a probabilistic inference method was used to identify voxels containing large arteries [26], and in such regions a separate intra-vascular component was included in the model. This technique appears effective for eliminating the vascular artefact in multi-TI ASL data, however, it assumes that the intra-vascular bolus passes instantaneously through the voxel, and is therefore best suited to modelling the intra-vascular signal in large vessels with high flow speeds. While this may be a reasonable assumption in healthy tissue, this may not be the case for the chaotic and tortuous vascular structure observed in tumors.

A number of studies have examined the use of ASL for measuring perfusion in brain tumors (eg. [11], [27]–[29]). However, the majority of these have acquired ASL data at a single TI, and as such the dynamic behaviour of the labelled bolus as it traverses the tumor vasculature could not be evaluated. We present here a study in which ASL was performed in a small cohort of paediatric brain tumor patients, with acquisitions made over a wide range of TIs, in order to probe perfusion kinetics in the tumor environment. Rather than treating the intra-vascular signal as an artefact, we developed a modified, ‘two-stage’ version of the Buxton general kinetic model, in which the total ASL signal in each voxel is divided into a non-exchanging, ‘pre-capillary’ stage, and a later, exchanging stage in the capillary bed. The model is similar in nature to [25], however, in this study modifications are made to the functional form of the tissue residue function described in [17], and the assumption of instantaneous passage of the intra-vascular bolus through the voxel is relaxed, which allows us to include smaller arteries and arterioles in the pre-capillary stage.

The two-stage model was first tested in healthy adult volunteers, in which multi-TI ASL data were acquired. This was followed by a magnetic resonance angiography (MRA) acquisition in two subjects, to test the hypothesis that the ASL-derived estimates of high intra-vascular signal corresponded to regions of large arterial vessels. We then applied the technique to ASL data collected in a cohort of paediatric brain tumor patients, to test the utility of the technique for measuring the dynamic perfusion signal in the presence of abnormal vascular structure. We also investigated whether parameter maps derived from this model offer a novel contrast in the tumor environment and surrounding tissue, which could provide surrogate biomarkers of both CBF and abnormal vascular structure, derived using dynamic ASL data alone.

## Materials and Methods

### Theory

The general kinetic model presented in [17] describes the dynamic ASL signal in terms of three time dependent basis functions. The first is the delivery function, *c(t)*, which represents the normalized arterial concentration of magnetization arriving at a voxel at time *t*. If the bolus of labelled blood arrives at time *t = BAT*, then the second basis function is the tissue residue function *r(t)*, which describes the fraction of tagged water molecules that remain in the tissue after arrival (*t>BAT*). The third basis function is the magnetization relaxation function, *m(t)*, which describes the fraction of the original longitudinal magnetization tag carried by the water molecules that remains at time *t*. It is assumed in [17] blood water instantaneously exchanges with tissue on arrival in the voxel, resulting in the following form of the basis functions (for pulsed ASL):
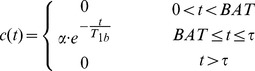
(1)

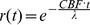
(2)

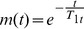
(3)where *α* = inversion efficiency of the tagging pulse, *T_1b_* = longitudinal relaxation time of arterial blood, *T_1t_* =  longitudinal relaxation time of tissue, *CBF* = cerebral blood flow, *λ* =  equilibrium tissue/blood partition coefficient of water, and Τ = bolus duration. The time dependent ASL signal is then:
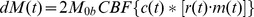
(4)where *M_0b_* is the equilibrium longitudinal magnetization of arterial blood and 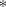 denotes convolution [17].

**Figure 1:**
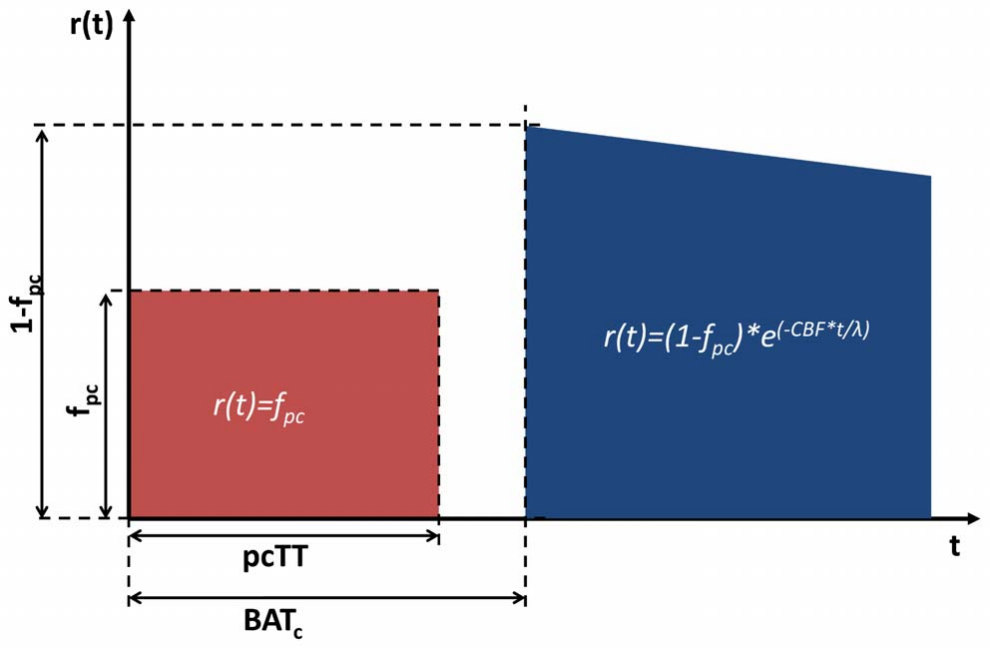
Illustration of the adapted tissue residue function r(t) used in the two-stage model. The ASL signal arises from two sources: the non-exchanging, ‘pre-capillary blood volume’ (volume fraction f_pc_), and the exchanging, capillary blood volume (volume fraction 1- f_pc_). Labelled blood traverses the pre-capillary stage during the ‘pre-capillary transit time’ (pcTT), and arrives at the capillary bed at the ‘capillary bolus arrival time’ (BAT_c_).

In this study we adapted the form of *r(t)*, to account for a pre-capillary stage (Figure 1). This two-stage model assumes that the total ASL signal in a given voxel arises partly from blood in the non-exchanging, pre-capillary blood volume (with volume fraction f_pc_), and partly from blood in exchanging capillaries (with volume fraction 1- f_pc_). Labelled blood resides in the pre-capillary stage during the ‘pre-capillary transit time’ (pcTT), and arrives at the capillary bed at the ‘capillary bolus arrival time’ (BAT_c_). The two stages are independent (i.e. the capillary stage does not have to directly follow the pre-capillary stage), to account for the fact that voxels may contain arteries/arterioles which terminate at capillaries outside of the voxel. This may be the case when a voxel contains either large through-flowing arteries, or tortuous vessels which may pass into and then out of the voxel. As such, *r(t)* represents a combined residue function from two independent sources of signal. Single compartment kinetics were assumed for the capillary stage to limit the number of free parameters in the model, and the T_1_ value used in *m(t)* was adapted for each stage, resulting in the following expressions for *r(t)* and *m(t)*:
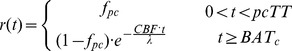
(5)

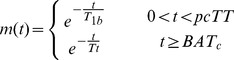
(6)
Equations (5) and (6) were substituted into equation (4) for the calculation of *dM(t)*.

### Simulated data

To provide an illustration of how different levels of f_pc_ influence both the total ASL signal, and the preferred model choice, a series of simulated ASL datasets were generated. These were produced by assuming the total ASL signal arises from two independent sources: a pre-capillary, intra-vascular source (stage 1), and a capillary-tissue exchanging source (stage 2). The signal from each source was generated independently using the single-stage model (equations 1–4), and the following parameters were used to generate the data: BAT_1_ = 0.1 s, Τ_1_ = 0.4 s, CBF_1_ = 50 ml/100 g/min, BAT_2_ = 0.9, Τ_2_ = 0.7 s, CBF_2_ = 50 ml/100 g/min, T_1t_ = 1.1 s, and T_1b_ = 1.3 s. These were chosen to be representative of a typical imaging voxel in gray matter (GM), which contains both capillaries and non-exchanging arteries/arterioles (the relative volume fraction of the latter being controlled via the f_pc_ parameter). Using these parameters, raw ASL datasets were created for a range of f_pc_ values (0.0–1.0), with the total signal (*dM_tot_*) being the weighted sum of the two stages: i.e. *dM_tot_* = (f_pc_ *dM_1_)+(1-f_pc_)*dM_2_. The synthetic ASL signal was then sampled at 12 TI times, ranging from 0.2 to 2.4 s in 0.2 s intervals, to match the *in vivo* ASL protocol (see Methods: *Imaging Protocol*). Following this, the single- and two-stage models were fit to the raw data, and the Bayesian Information Criteria value (see Methods: *Post Processing*) was recorded for each model.

### Participants

#### Ethics Statement

Approval to conduct this study was obtained from the Research Ethics Committee at Great Ormond Street Hospital. All healthy adults imaged for this study provided written informed consent, in accordance with our institutional ethical review board. In the case of paediatric patients, verbal consent was provided by the patients themselves, and written consent was also provided by a parent/guardian.

All *in vivo* experiments were performed on a 1.5 T Siemens Magnetom Avanto scanner (Siemens, Erlangen, Germany), equipped with 40 mT/m gradients and a 12 channel head receive coil. Eight healthy adults (mean age = 29 years) and 8 paediatric brain tumor patients (mean age  = 11.5 years) were imaged. The brain tumor cohort consisted of the following tumor types (with histologically determined tumor grade according to the World Health Organization (WHO) classification shown in brackets): 4 pilocytic astrocytomas (all grade I), 3 gangliogliomas (2x grade I, 1x grade III), and 1 glioblastoma multiforme (grade IV).

### Imaging Protocol

ASL data were acquired using a flow-sensitive alternating inversion recovery (FAIR) pulsed-ASL sequence, with 3D single shot GRASE data acquisition [16], with the following imaging parameters: TR = 3.0 s, TE = 31.6 ms, NSA = 8, field of view (FOV)  = 230 mm, matrix size = 64×64, 20 contiguous slices with 5 mm thickness. Measurements were made at 12 inflow times (TI), ranging from 0.2 to 2.4 s in 0.2 s intervals, with total scan time = 9.6 min. Background suppression of static tissue was used, as described in [16], with x10 scanner gain, to maximise the signal from the inflowing blood. A series of inversion recovery acquisitions (TI = 0.2, 0.6, 1.4, 2.4 s) with identical readout, FOV and resolution were also acquired without background suppression, for quantification of *T_1t_* and *M_0t_* in each voxel.

In every subject, a T2-weighted acquisition (TR/TE = 3800/120 ms) was made with identical FOV and resolution as the ASL scan, to aid in tissue segmentation. Two healthy adults also received a time of flight MRA acquisition, with 70 slices of 0.9 mm thickness, in-plane resolution of 0.49×0.49 mm, flip angle of 20°, TR = 36 ms, TE = 6.3 ms. In the paediatric patients, ASL was added to the standard clinical brain tumor imaging protocol in use in our institution, which includes a three-dimensional fast low angle shot (FLASH) sequence, with flip angle = 15°, TR = 11 ms, TE = 4.94 ms, voxel size = 1 mm isotropic, slices = 176, and T1-weighted acquisitions pre and post-contrast (Dotarem, 0.2 ml/kg).

### Post Processing

All data analysis was performed using Matlab (MathWorks Inc., Natick, MA), and all model fitting was performed using an iterative Nelder-Mead nonlinear least squares algorithm. Firstly, voxel-wise values of *T_1t_* and *M_0t_* were calculated by fitting the signal intensity from the inversion recovery acquisitions to the following equation:
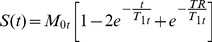
(7)with *T_1t_* and *M_0t_* as fitted parameters. Values of *M_0b_* were calculated on a voxel wise basis (to account for B_1_ inhomogeneities), using *M_0b_ = M_0t_/λ*, with *λ* = 0.9 [30].

The two-stage ASL model (equation 4) was then fit to the measured *dM* values in each voxel, with the modified versions of *r(t)* and *m(t)* described in equations (5) and (6) incorporated. The fitted parameters were *BAT, CBF, pcTT*, *f_pc_*, and *BAT_c_*, with Τ fixed at 0.7 s (based on previous studies performed using the same ASL protocol at our institution [31]), and α = 1.0. The single-stage Buxton general kinetic model [17] was also fit to the *dM* values, with *BAT* and *CBF* as fitted parameters (again with Τ fixed at 0.7 s), and the two models were compared using the Bayesian Information Criterion (BIC):
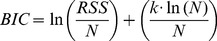
(8)where *RSS* = residual sum of squares between fitted and measured *dM* values, *N* = number of TIs (*N* = 12), and *k* = number of free parameters (*k* = 5 in the two-stage model, *k* = 2 in the single-stage model). Values of BIC were calculated on a voxel-wise basis, with the model producing the lower value being the preferred model in that voxel.

Whole tumor regions of interest (ROIs) were manually drawn on the T2-weighted images acquired during the imaging session, obtained using an identical FOV and voxel resolution as the ASL acquisition. Tumor ROIs were identified as hyper-intense regions on the T2w image, with the routine clinical images (e.g. post-gadolinium T1-weighted) used to guide tracing of the tumor outline. In each patient, a ‘healthy tissue’ ROI was also defined. For tumors located laterally, this was defined as an ROI of equal volume, contralateral to the tumor. For midline tumors, the healthy tissue ROI was defined as a region of equal volume, drawn in the GM lateral to the tumor region. In the healthy adults, whole-brain GM masks were obtained by automatic segmentation of the T2-weighted images, using the FAST module within FSL (FMRIB’s Software Library, Oxford University, www.fmrib.ox.ac.uk/fsl). In every subject (patients and healthy controls), a whole-brain mask was also obtained using the BET module within FSL. Noise regions were defined as all voxels outside of this mask. A noise filter was then applied to all raw ASL data prior to processing, to exclude voxels in which the peak *dM* value was less than 3 times greater than the mean *dM* value in the noise, to exclude regions with very low perfusion (e.g. necrotic and highly oedematous regions).

In previous clinical studies involving dynamic susceptibility contrast enhanced perfusion MRI (DSC MRI), the most widely used parameter for predicting tumour grade has been tumour vascularity, defined as the relative cerebral blood volume (rCBV) [32]–[35]. This parameter can be derived from the central volume theorem [36], which states:
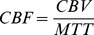
(9)where MTT = mean transit time. The aim of this study was to introduce a novel two-stage model for describing perfusion kinetics, which is able to describe biphasic behaviour in the ASL signal in both healthy and tumour tissue: as such, the patient cohort we demonstrated this in was not large enough for a statistical comparison between different tumour subtypes / grades. Nonetheless, it is worth examining if the derived parameters from the two-stage model show potential for predicting tumour grade in future clinical studies. Therefore, following previous DSC MRI studies, we aimed to measure tumour vascularity in our clinical cohort. Although the f_pc_ parameter is derived from the two-stage model, this represents a weighting fraction on the total ASL signal which arises from the pre-capillary vasculature, and does not reflect the pre-capillary blood volume in physiological units (e.g. ml/100 g tissue). However, using equation 9, it is possible to define an equivalent CBV parameter in such units, by multiplying the fitted values of CBF and pcTT from the two-stage model. We henceforth refer to this parameter as pcCBV, with units of ml blood /100 g tissue. Previous DSC studies have shown improved stratification of tumour grade and reproducibility when measurements are made in areas of maximum abnormality in rCBV maps [37], [38]. We followed a similar approach here, and defined an automatically generated ROI within each tumour, based on voxels which demonstrated pcCBV above the 90^th^ percentile value within the tumour. Mean values of all fitted parameters were recorded in this ROI in each lesion.

## Results

### Simulated Data

**Figure 2:**
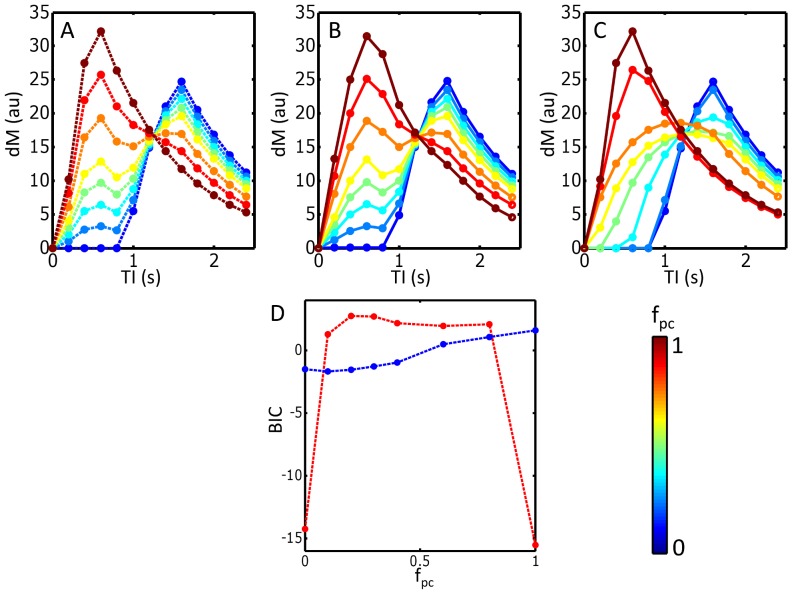
Results from the analysis of simulated ASL datasets, containing both a pre-capillary component (initial peak in dM curve around TI = 0.6 s) and an exchanging capillary component (second peak in dM curve, around TI = 1.6 s). (A) Simulated raw ASL data, for f_pc_ values ranging between 0 and 1 (see color bar), with the total signal sampled at 12 TI times (filled circles). The fits to the raw data from the two-stage and single-stage models are shown in (B) and (C) respectively. (D) Bayesian information criteria (BIC) value from fitting the single-stage (red) and two-stage (blue) models, as a function of f_pc_. The model producing the lower BIC value is the preferred model.

The results from the analysis of the simulated data are shown in Figure 2. When f_pc_ = 0 or 1, the simulated data represent a voxel containing only exchanging capillaries or non-exchanging arteries respectively, and therefore no bi-phasic behaviour is seen in the raw data. In these cases, the ASL signal was accurately described by the single stage model, and the BIC values from this model were correspondingly much lower than those from the two-stage model, due to the smaller number of fitted parameters. When biphasic behaviour was seen in the raw data (0<f_pc_<1), the single stage model was unable to accurately describe the raw ASL data, and the BIC values from the two-stage model were lower than those from the single stage model, suggesting that this is the preferred model. In this range of f_pc_ values, the mean fitted value of CBF from the single stage model was significantly underestimated (32±14 ml/100 g/min), compared to the mean CBF value from the two-stage model (58±4 ml/100 g/min; note the underlying value of CBF used to generate the raw data was 50 ml/100 g/min).

### Two-stage model validation in healthy adults

**Figure 3:**
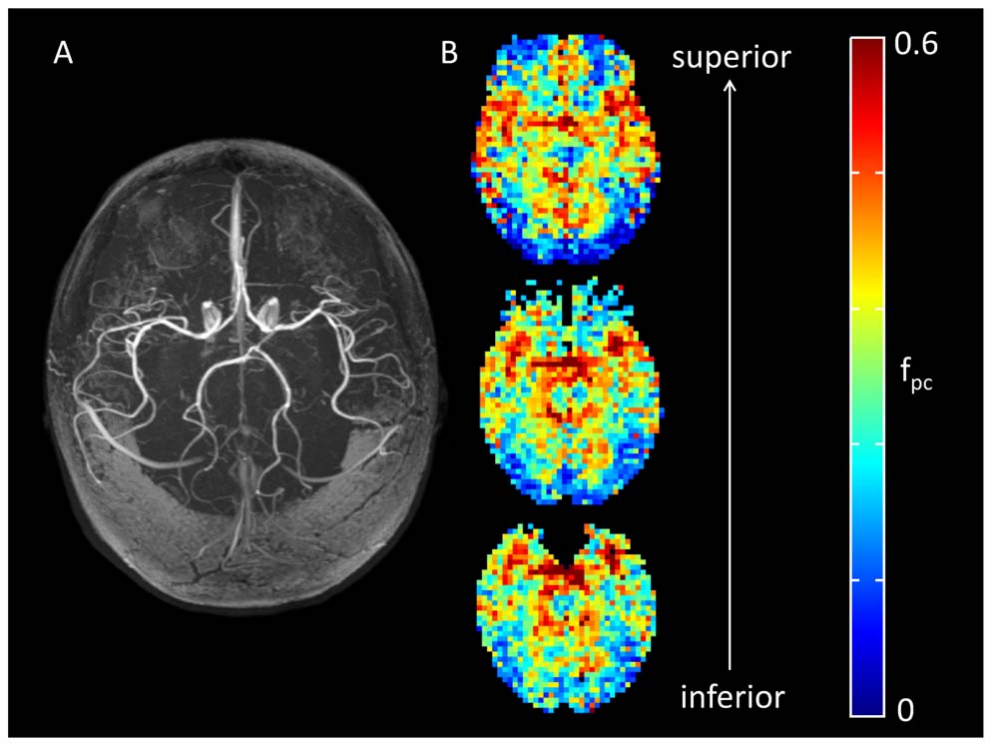
Comparison of time of flight angiography data and two-stage ASL model fitting in a healthy adult. (A) Axial maximum intensity projection from time of flight angiography, showing large arteries such as the anterior, middle, and posterior cerebral arteries. (B) Maps of the fitted f_pc_ parameter (pre-capillary blood volume), derived from ASL data collected in the same subject.

A comparison between the large arterial vessels identified using time of flight angiography, and fitted values of f_pc_ from the two-stage ASL model, is illustrated for a representative healthy adult in Figure 3. Voxels in which a large fraction of the total ASL signal originates from arteries or arterioles appear bright on f_pc_ maps; in both subjects the location of these voxels showed good visual agreement with the position of the larger arteries (e.g. anterior, middle, and posterior cerebral arteries) identified in the MRA acquisition.

Using the two-stage model, the mean voxel-wise CBF in the GM across the healthy adult cohort was 76±3 ml/100 g/min (mean±SD), compared to 67±4 ml/100 g/min using the single-stage model. Mean values of f_pc_, *pcTT* and *BAT_c_* were 0.34±0.03, 894±25 ms and 705±19 ms respectively. Across all healthy subjects, 37±4% of voxels in the GM were best described by the two-stage model.

### Brain Tumor Cohort

**Figure 4:**
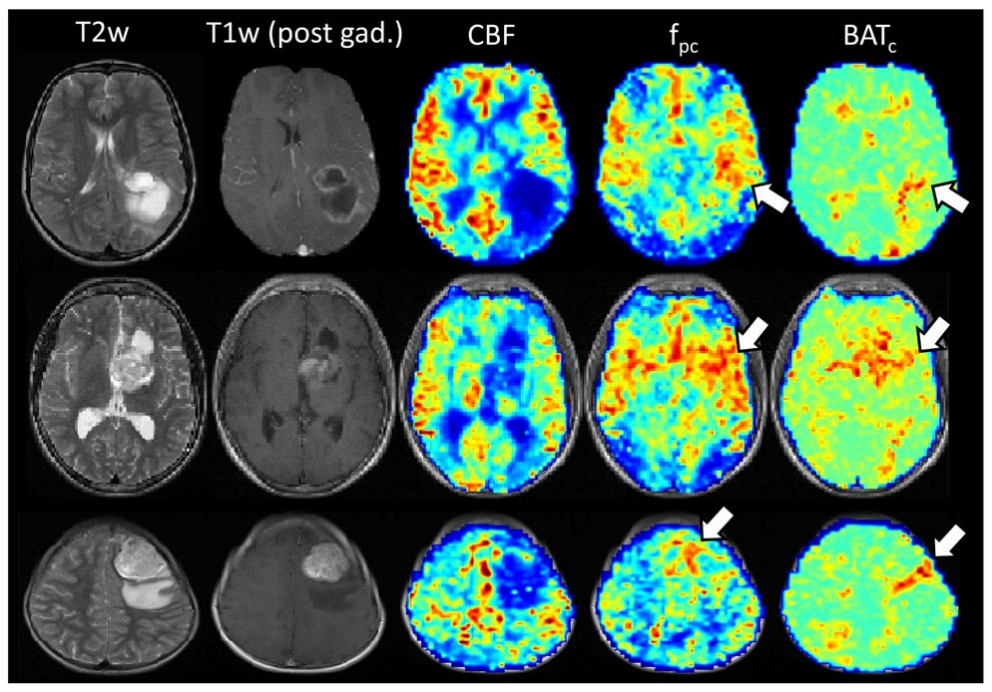
Examples of standard T2w and T1w (post contrast) images obtained in three brain tumor patients, with fitted values of cerebral blood flow (CBF), pre-capillary blood volume (f_pc_) and bolus arrival time at the capillary bed (BAT_c_) overlaid. The top row shows data from a 10 year old male with a glioblastoma multiforme (WHO grade IV). The middle row shows data from a 15 year old male with a ganglioglioma (WHO grade I), and the bottom row is from a 7 year old female with an anaplastic ganglioglioma (WHO grade III). The white arrows highlight regions of increased f_pc_ and BAT_c_ in the tumor region. Fitted parameter maps are shown after affine registration to the FLASH images acquired during the same imaging session.

**Figure 5:**
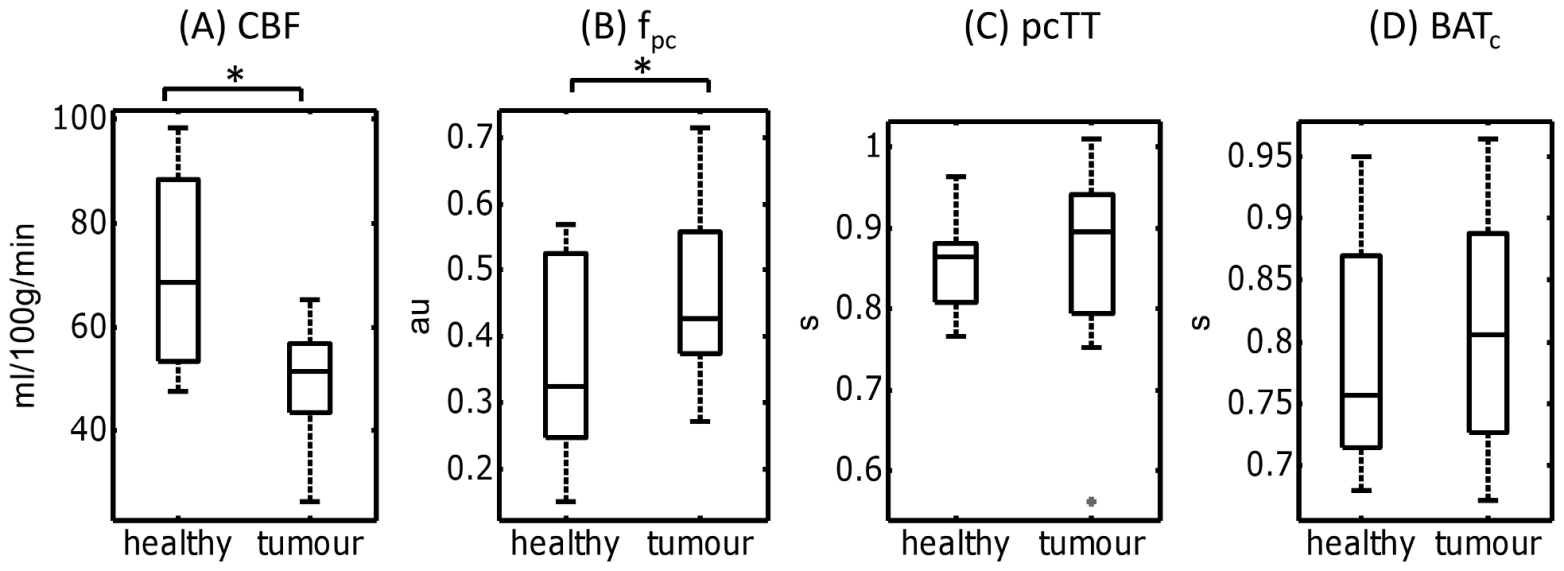
Comparison of fitted parameters from the two-stage ASL model in tumour tissue and contralateral healthy gray matter. Box and whisker plots of mean fitted values of (A) cerebral blood flow (CBF), (B) pre-capillary blood volume (f_pc_), (C) pre-capillary transit time (pcTT) and (D) bolus arrival time at the capillaries (BAT_c_). Data are shown for healthy tissue and tumor regions in all patients, with horizontal lines  =  group median, box edges  = 25th and 75th percentiles, and outliers (+) defined as values larger than q3+1.5*(q3−q1) or smaller than q1−1.5*(q3−q1). Significant difference between groups, determined using a two-tailed paired *t* test, is indicated by * (p<0.05).

Standard images (T2w and post-contrast T1w), and example maps of *CBF*, f_pc_ and *BAT_c_* from the two-stage model, are shown after affine registration to the FLASH images acquired during the same imaging session, for three representative patients, in Figure 4. Regions of elevated f_pc_ and BAT_c_ were observed in and around the tumor regions, as indicated by the white arrows in Figure 4. A comparison between the mean values of the fitted parameters derived from the two-stage model, from whole-tumor ROIs and contralateral healthy tissue and in all patients, is shown in Figure 5.

In contralateral healthy tissue, the number of voxels best described by the two-stage model (according to the BIC values) was 40±22% (similar to the mean GM value in the healthy adult cohort). This increased significantly in tumor tissue, to 52±15% (p<0.05). Median cerebral blood flow was lower in the tumor environment compared to contralateral healthy tissue (52±12 ml/100 g/min in tumor tissue, compared to 69±19 ml/100 g/min in contralateral healthy tissue, p<0.01, two-tailed paired *t* test). There was a significant increase in the median f_pc_ in tumor tissue compared to contralateral healthy tissue (0.43±0.14 vs. 0.32±0.16, p<0.05). There was a non-significant increase in both pcTT and BAT_c_ in tumor tissue, compared to healthy tissue (895±141 vs 863±63 ms, and 806±102 vs 757±99 ms respectively, p>0.05).

**Figure 6:**
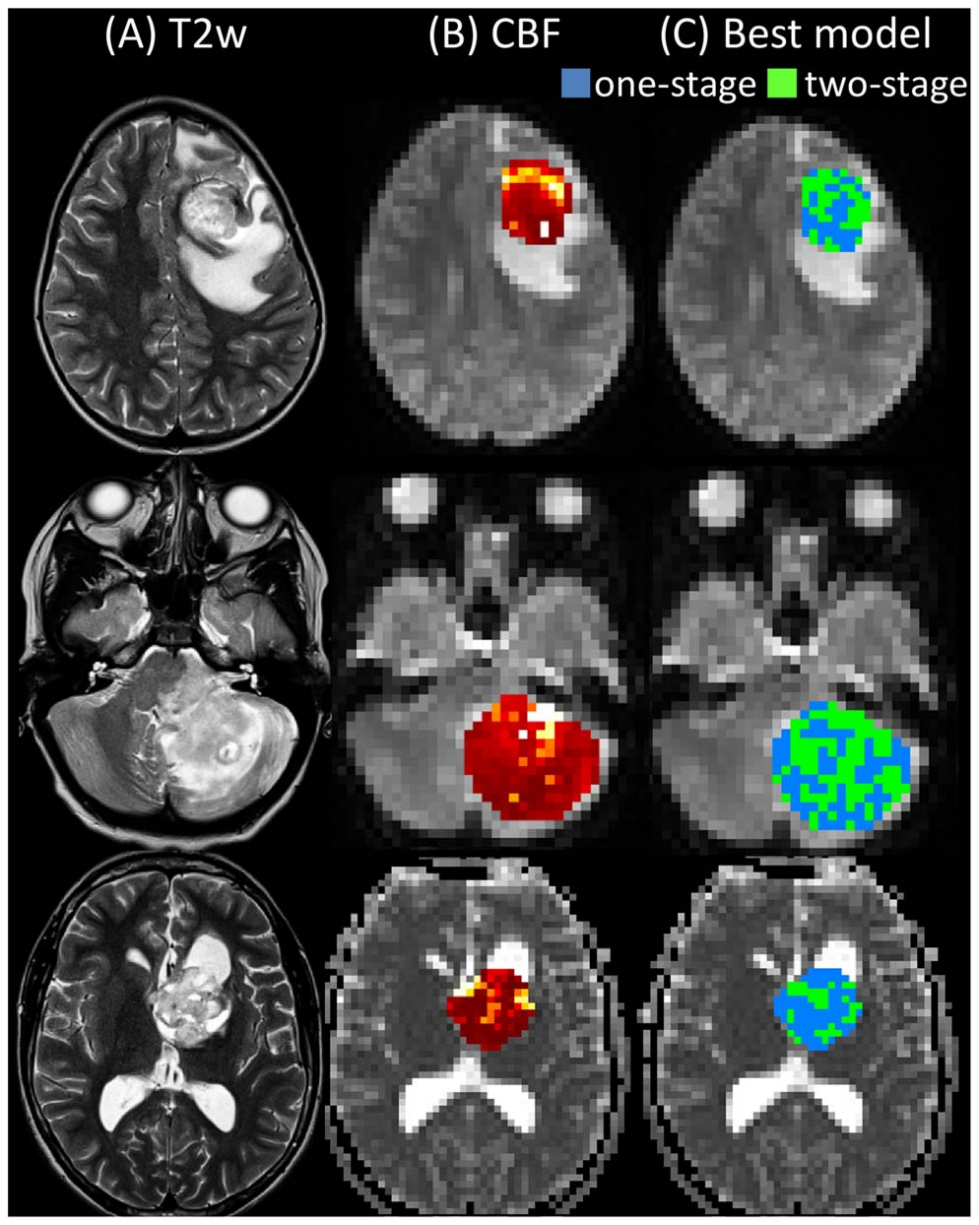
Regional variations in optimum model choice within the tumour environment. Examples of (A) T2w standard clinical images, (B) T2w images acquired at the native resolution of the ASL acquisition, with fitted CBF maps from the two-stage model overlaid in the tumor region, and (C) regional variations in the optimum model choice within the tumor region. Voxelwise BIC values were calculated for the single-stage and two-stage models, with the model producing the lower BIC value being the preferred model in that voxel (blue voxels  =  single-stage preferred, green voxels  =  two-stage preferred). Images from three representative patients are shown: the top row is from a 7 year old female with an anaplastic ganglioglioma (WHO grade III), the middle row is from an 10 year old female with a pilocytic astrocytoma (WHO grade I), and the bottom row is from a 15 year old male with a ganglioglioma (WHO grade I).

**Figure 7:**
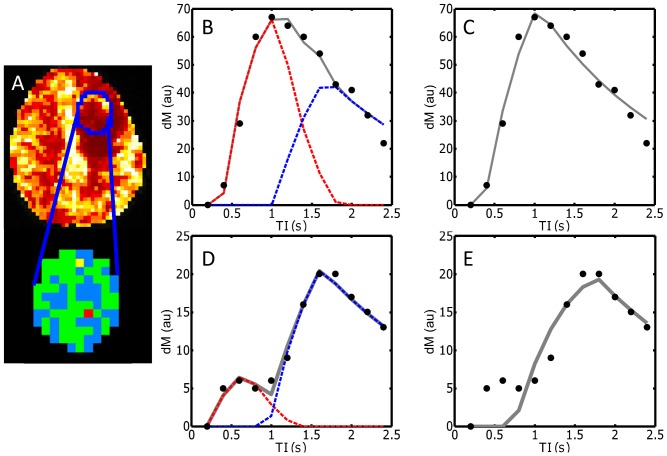
Examples of raw ASL data, and model fitting within the tumor ROI, in one patient (ganglioglioma, grade III). A map of fitted CBF values, and regional variations in the optimum model choice within the tumor region (outlined in blue), are shown in A (blue voxels  =  single-stage preferred, green voxels  =  two-stage preferred). Raw and fitted data from two example voxels are shown in B–E. The yellow voxel in A represents a region in which the single-stage model is preferred – the raw data from this voxel are represented by the filled circles in B and C. The gray lines in B and C represent the fitted values from the two-stage and single-stage models respectively. The red dashed lines represent the modelled contribution from the pre-capillary stage, and the blue dashed lines represent the contribution from the capillary stage. The red voxel in A represents a region in which the two-stage model is preferred, and the raw data from this voxel, and the fits from the two-stage and single-stage model, are shown in D and E respectively.

Example maps of CBF and regional differences in the optimum model choice within a lesion are shown for three representative patients in Figure 6. An example of raw ASL data, and the fits from the single- and two-stage ASL models, are shown for two different regions within a tumor, for a representative patient in Figure 7.The plots show examples of both mono-phasic and biphasic raw ASL data. The mono-phasic example originates from a voxel placed within a large artery which runs into the tumor region. The ASL signal in this region is dominated by the high CBF in this artery, and there is therefore little evidence of biphasic behaviour, and the single-stage model is preferred. The biphasic example originates from a voxel within the tumor, but more distal to the large artery. The ASL data appears biphasic here, and the two-stage model is preferred.

**Figure 8:**
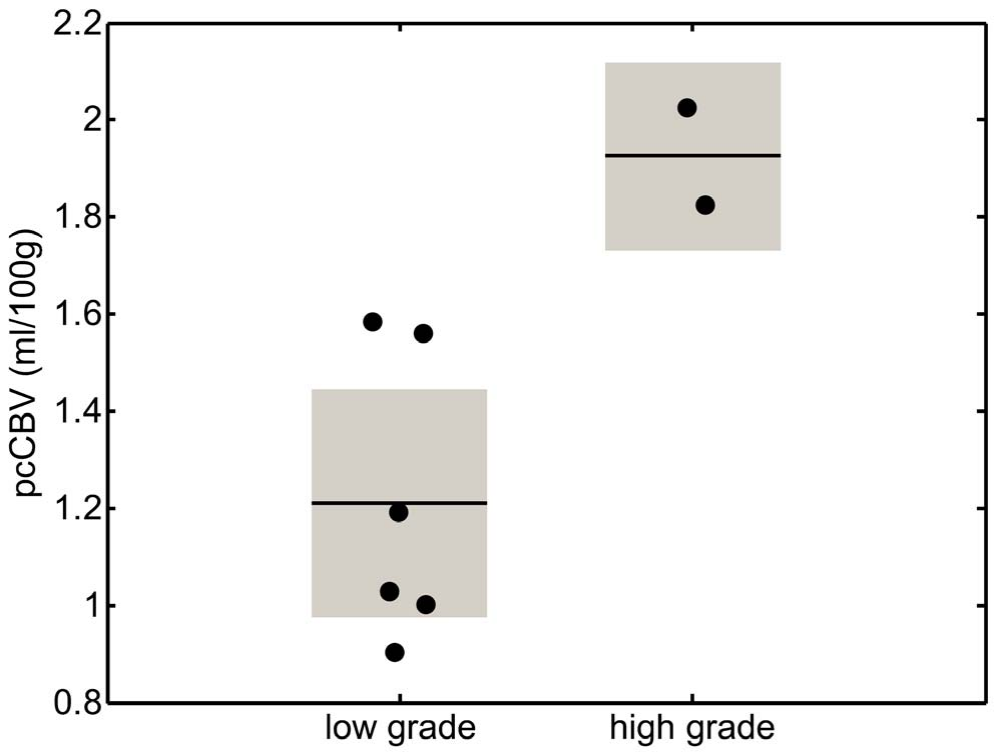
Mean values of pcCBV in the low- and high-grade lesions included in this study. Filled circles represent the mean value from a given patient, based on an ROI placed in regions of high pcCBV in the lesion (see Methods: *Post Processing*). Solid lines represent the group mean, and shaded boxes represent the standard deviation.

Mean fitted values from both models (single- and two-stage) in the automatically generated tumour ROIs (based on regions of high pcCBV in each lesion, see Methods: *Post Processing*), are shown for each patient in Table 1. The best discrimination between low grade (WHO I-II) and high grade (WHO III-IV) lesions was found in the pcCBV parameter, as illustrated in Figure 8. However, due to the small numbers of patients included in this study (particularly in the high grade group), no statistical comparisons were made between the two groups.

**Table 1:** Fitted parameters from the single- and two-stage model in all patients.

			Single-stage	Two-stage				
ID	Subtype	WHO grade	CBF	CBF	f_pc_	pcTT	BATc	pcCBV
			(ml/100 g/min)	(ml/100 g/min)	a.u.	s	s	ml/100 g
1	pilocytic astrocytoma	I	79	65	0.14	0.83	0.70	0.90
2	pilocytic astrocytoma	I	48	39	0.49	1.64	0.92	1.03
3	pilocytic astrocytoma	I	101	93	0.38	1.06	0.68	1.56
4	pilocytic astrocytoma	I	127	68	0.13	0.91	0.78	1.00
5	ganglioglioma	I	73	83	0.74	1.28	0.88	1.58
6	ganglioglioma	I	54	54	0.47	1.49	0.80	1.19
7	ganglioglioma	III	110	122	0.60	1.02	0.71	2.02
8	glioblastoma multiforme	IV	113	105	0.54	1.16	0.73	1.83

## Discussion

### Simulated Data

The simulated data show that, as the bi-phasic nature of the ASL signal starts to increase, the ability of the single-stage model to describe the data decreases. This pattern continues until eventually the total ASL signal is dominated by the pre-capillary stage (for f_pc_>∼0.8), after which the signal begins to look mono-phasic once more. When biphasic behaviour is prominent, the resulting fitted CBF values from the single-stage model are underestimated. The reason for this is that the single-stage model cannot capture the ‘dual peaks’ seen in the raw data, and therefore as f_pc_ increases, the fitted curve from the single-stage model becomes ‘wider’ and ‘flatter’ in an attempt to minimize the least-squares error between the raw data and modelled values (see Figure 2, panel C). This results in lower fitted values of CBF (which is proportional to the height of the peak of the fitted *dM* curve). Because the two-stage model is able to account for the ‘dual peaks’ seen in biphasic raw ASL data, fitted CBF values are not underestimated in the same way, and remain closer to the true underlying value.

### In vivo Data: Healthy Adults

The good agreement between the location of large arteries identified using MRA in the healthy volunteers, and regions of elevated f_pc_ derived from the ASL data, suggests that the two-stage model is effective at identifying voxels in which a large proportion of the ASL signal arises from the pre-capillary stage.

There is a considerable range in previously reported values of CBF in healthy adult GM, with average values of 77 ml/100 g/min from ASL methodologies, 69 ml/100 g/min from DSC-MRI, and 55 ml/100 g/min from PET ([39] and references therein). The two-stage model produced a mean voxel-wise value of CBF in our healthy subject's GM which agrees well with previous ASL studies (76 ml/100 g/min). The reduced value from the single-stage model (67 ml/100 g/min) is due in part to the mean f_pc_ value of 0.34 found in healthy GM; the results from the simulated data suggest that the data will appear biphasic at this level of f_pc_, and therefore CBF values from the single-stage model will be underestimated. The mean f_pc_ value in healthy GM suggests that a significant proportion of voxels in this region suffer partial volume effects, and contain signal from both the capillary bed and larger arteries / arterioles. This is perhaps not surprising, given vascular crushing gradients were not applied, the large size of our imaging voxels, and the close proximity of large arterial vessels to many of the voxels in the GM.

### In Vivo Data: Brain Tumor Patients

Overall, there was a significant increase in the number of voxels displaying biphasic behaviour in the tumor environment (*cf*. contralateral healthy tissue), with over half of all tumor voxels being best described by the two-stage model. In a number of lesions, the regions of biphasic behaviour tended to ‘branch off’ from areas of high CBF (which are likely to be large arteries, see Figure 6), which suggests an abnormal vascular structure stems from larger arteries feeding into the tumor environment.

The increase in f_pc_ in tumor tissue implies the pre-capillary contribution to the ASL signal is increased, which perhaps reflects increased vascularity in the tumour environment. There was also trend towards increased values of pcTT and BAT_c_ in tumour tissue, which suggests that labelled blood-water spends a longer time traversing this vasculature, although these fell short of significance. It is worth re-iterating that the two stages are independent in our two-stage ASL model, and as such pcTT and BAT_c_ are not required to be equal. In fact, in general BAT_c_ was slightly lower than pcTT, in both healthy and tumor tissue. This suggests that some of the pre-capillary signal in a given voxel arises from arteries / arterioles which do not feed directly into the capillary bed within that voxel.

The marked increase in pcCBV in the high grade tumours suggests that this parameter, which combines fitted values of CBF and pcTT, may be the most useful in separating low and high grade lesions in future clinical studies. This parameter is sensitive to both changes in blood flow in the tumour environment, and increased tortuosity of the blood vessels (which would increase pcTT), which may explain the increased sensitivity of this parameter to tumour grade.

Overall, these findings support the assertions made earlier: that new vasculature is formed to feed a tumor, and these vessels tend to be more dilated and/or tortuous than those seen in healthy tissue. Furthermore, the increased tortuosity means it is more likely that pre-capillary vessels will pass into and out of the imaging voxel before terminating in capillary exchange sites (which may be outside of the imaging voxel), which would contribute to the increased level of biphasic behaviour seen in dynamic ASL data acquired in the tumor environment.

### Study Limitations

As a rather heterogeneous cohort of patients was used to examine perfusion dynamics in this study, it was not possible to determine whether significant differences in the modelled parameters exist between tumor subtypes and grades, and future clinical studies are needed to explore this. Instead, this cohort of patients was chosen to provide a testing ground for the investigation of the biphasic ASL signal in tumors, and to illustrated the application of the two-stage model.

Secondly, future studies are needed to explore the direct histopathological correlation between the fitted parameters from the two-stage model, and the properties of the tumour vasculature. This would help to clarify any ambiguities in the biophysical explanation of the fitted parameters – for instance, regions of increased f_pc_ in the tumour could be due to either neoangiogenesis, or vasodilation, and it is difficult to separate these processes with ASL data alone.

Thirdly, for tumors bordering the cerebral cortex or subarachnoid space, partial volume effects with the leptomeningeal arteries, which are vigorously perfused in children, may increase the fitted f_cp_ parameter. In our cohort, abnormalities in the f_cp_ maps did not appear focussed on these regions, however, this could represent a source of error in future studies.

Also, although the simulated *dM* curves presented in Figure 2 are similar in form to the experimental data obtained in the tumor environment (Figure 7), it is difficult to generate synthetic data that accurately reflects the expected ASL signal in the haphazard tumor vascular network. The simulated data are therefore presented here as a simplified illustration of how biphasic behaviour in the ASL signal can cause errors in model fitting and in the estimation of CBF when using a single-stage model.

Lastly, although we fit both the single- and two-stage models in each voxel in this study, to demonstrate regional variations in optimum model choice, in practice only the optimum model should be fit in each voxel. This could be performed on a post-hoc basis, in which data from the two-stage model are disregarded in voxels where the single stage model is preferred (based on BIC values). Alternatively, a probabilistic inference method similar to that presented in [25] could be used to determine voxels in which the two-stage model should be used.

### Conclusion

We have demonstrated that dynamic ASL data acquired in the tumor environment displays a significantly increased level of biphasic behaviour compared to healthy tissue, and we present here a two-stage model to describe this behaviour. As illustrated in Figure 4, maps of the fitted parameters obtained using the two-stage model offer a novel contrast in the tumor environment and surrounding tissue, which, if the hypotheses described above are correct, would allow surrogate biomarkers of both CBF and abnormal vascular structure to be derived from dynamic ASL data. This could provide a useful tool for *in vivo* assessment of tumor malignancy. It has been shown that vessel shape changes do not correlate directly with CBF measurements [40], and therefore an ASL model which is sensitive to both CBF and vascular structure could provide valuable information in studying tumor development. Also, as vessel shape rapidly normalizes during successful cancer treatment [41], the non-invasive nature of this technique would make it a useful methodology for longitudinal monitoring of treatment response to anti-angiogenic therapies in future patients.

## Acknowledgments

The authors would like to thank Tina Banks for radiological support, and the patients and healthy volunteers who were imaged as part of this study.
